# Transmission Bottleneck Size Estimation from Pathogen Deep-Sequencing Data, with an Application to Human Influenza A Virus

**DOI:** 10.1128/JVI.00171-17

**Published:** 2017-06-26

**Authors:** Ashley Sobel Leonard, Daniel B. Weissman, Benjamin Greenbaum, Elodie Ghedin, Katia Koelle

**Affiliations:** a Department of Biology, Duke University, Durham, North Carolina, USA; b Department of Physics, Emory University, Atlanta, Georgia, USA; c Tisch Cancer Institute, Departments of Medicine, Oncological Sciences, and Pathology, Icahn School of Medicine at Mount Sinai, New York, New York, USA; d Center for Genomics and Systems Biology, Department of Biology, and College of Global Public Health, New York University, New York, New York, USA; Wake Forest University

**Keywords:** bottleneck, influenza A virus, next-generation sequencing

## Abstract

The bottleneck governing infectious disease transmission describes the size of the pathogen population transferred from the donor to the recipient host. Accurate quantification of the bottleneck size is particularly important for rapidly evolving pathogens such as influenza virus, as narrow bottlenecks reduce the amount of transferred viral genetic diversity and, thus, may decrease the rate of viral adaptation. Previous studies have estimated bottleneck sizes governing viral transmission by using statistical analyses of variants identified in pathogen sequencing data. These analyses, however, did not account for variant calling thresholds and stochastic viral replication dynamics within recipient hosts. Because these factors can skew bottleneck size estimates, we introduce a new method for inferring bottleneck sizes that accounts for these factors. Through the use of a simulated data set, we first show that our method, based on beta-binomial sampling, accurately recovers transmission bottleneck sizes, whereas other methods fail to do so. We then apply our method to a data set of influenza A virus (IAV) infections for which viral deep-sequencing data from transmission pairs are available. We find that the IAV transmission bottleneck size estimates in this study are highly variable across transmission pairs, while the mean bottleneck size of 196 virions is consistent with a previous estimate for this data set. Furthermore, regression analysis shows a positive association between estimated bottleneck size and donor infection severity, as measured by temperature. These results support findings from experimental transmission studies showing that bottleneck sizes across transmission events can be variable and influenced in part by epidemiological factors.

**IMPORTANCE** The transmission bottleneck size describes the size of the pathogen population transferred from the donor to the recipient host and may affect the rate of pathogen adaptation within host populations. Recent advances in sequencing technology have enabled bottleneck size estimation from pathogen genetic data, although there is not yet a consistency in the statistical methods used. Here, we introduce a new approach to infer the bottleneck size that accounts for variant identification protocols and noise during pathogen replication. We show that failing to account for these factors leads to an underestimation of bottleneck sizes. We apply this method to an existing data set of human influenza virus infections, showing that transmission is governed by a loose, but highly variable, transmission bottleneck whose size is positively associated with the severity of infection of the donor. Beyond advancing our understanding of influenza virus transmission, we hope that this work will provide a standardized statistical approach for bottleneck size estimation for viral pathogens.

## INTRODUCTION

Infectious disease transmission relies on the transfer of a pathogenic organism from one host to another. This transfer is characterized by a transmission bottleneck, defined as the size of the founding pathogen population in the recipient host. Accurate quantification of transmission bottleneck sizes for pathogenic organisms is critical for several reasons. First, bottleneck sizes impact levels of genetic diversity in recipient hosts and thereby impact the rate at which pathogens can adapt to host populations, with smaller bottleneck sizes decreasing rates of adaptation (1, 2). Second, when cooperative interactions occur within a pathogen population (e.g., see references 3 and 4) or when viral complementation and cellular coinfection are critical for producing viral progeny (e.g., see reference 5), bottleneck sizes will necessarily impact initial pathogen replication rates, with larger bottleneck sizes enabling the occurrence of these interactions and thus facilitating within-host replication. Finally, transmission bottleneck sizes impact the ability to accurately reconstruct who infected whom during an ongoing epidemic (6), such that estimation of the transmission bottleneck size can point to cases which may be problematic and for which a certain class of phylodynamic inference methods (see reference 7) might be particularly useful.

The transmission bottleneck size has been estimated for a number of pathogenic organisms, including pathogens of plants (8–13) and animals (14–22). While those estimates relied on the distribution of pathogen types in infection recipients, as determined by molecular and phenotypic markers or Sanger sequencing of the pathogen population in donor and recipient hosts, deep-sequencing data have recently started to be used to gauge transmission bottleneck sizes (23–29). Some of those studies characterized the general magnitude of transmission bottleneck sizes, with results indicating that narrow, selective bottlenecks tend to govern the transmission dynamics of viral pathogens that are ill adapted to their recipient hosts (24–26). Studies that instead gauged transmission bottleneck sizes of well-adapted viral pathogens using deep-sequencing data have, in contrast, generally found that they tend to be loose, with many virions initiating infection (23, 28, 29). While many of those studies focus on assessing how “loose” or “narrow” a transmission bottleneck is, other studies have attempted to quantitatively estimate transmission bottleneck sizes. One approach relied on the use of barcoded influenza virus during experimental transmission studies in small mammals, with results indicating that the route of transmission greatly impacts the size of the bottleneck (27).

In natural infections, it is not feasible to rely on barcoded or otherwise marked pathogens. In these cases, statistical approaches have therefore instead been used to quantify bottleneck sizes (28, 30). Two studies have used the Kullback-Leibler divergence index (developed in reference 30) to estimate the viral effective population size initiating infection from deep-sequencing data (28, 30). One of those studies quantified the transmission effective population size for Ebola virus in human-to-human infections (30). The other study quantified this transmission effective population size for human influenza A viruses (IAVs) (28). A second statistical approach used previously (28) makes use of a single-generation population genetic Wright-Fisher model to estimate the effective viral population size initiating infection. While this approach similarly showed that the effective population size following influenza virus transmission in natural human-to-human infection is large, this model yielded quantitatively different results from those of the Kullback-Leibler approach. Furthermore, in both of those studies, it is not clear how the effective population size relates to the transmission bottleneck size. It is worth noting, however, that the effective population size is generally considered to be an underestimate of the true population size, as it represents the minimum population size necessary to establish observed levels of genetic diversity.

Both of these approaches (28, 30) analyze only variants that are identified as being present in both the donor and the recipient. However, the absence of a donor variant in a recipient host is also informative, and ignoring such missing variants can significantly bias transmission bottleneck size estimates. Another limitation of both approaches is that they do not consider the effect that stochastic dynamics early in infection may have on variant frequencies in the recipient. To address these concerns, here, we introduce a new method for estimating the transmission bottleneck size of pathogens. This method accounts for stochastic dynamics occurring during viral replication in the recipient and further accounts for variant calling thresholds that are used in calling a variant present or absent in a sample. In addition, this method has the ability to estimate a bottleneck size for individual transmission pairs. We refer to this method as the beta-binomial sampling method, based upon this method's derived likelihood expression. Using a simulated data set, we compare the beta-binomial sampling method to two methods of bottleneck size inference that are present (in some form) in the literature: the presence/absence method and the binomial sampling method. This comparison demonstrates that the beta-binomial sampling method is able to recover the true bottleneck size of the simulated data set, whereas the 2 other methods infer biased estimates by failing to account for variant calling thresholds or stochastic dynamics in the recipient host. Finally, we apply the beta-binomial sampling method to an existing next-generation sequencing (NGS) data set of influenza A virus infections to estimate the transmission bottleneck size in natural human-to-human flu transmission.

### Models.

**FIG 1:**
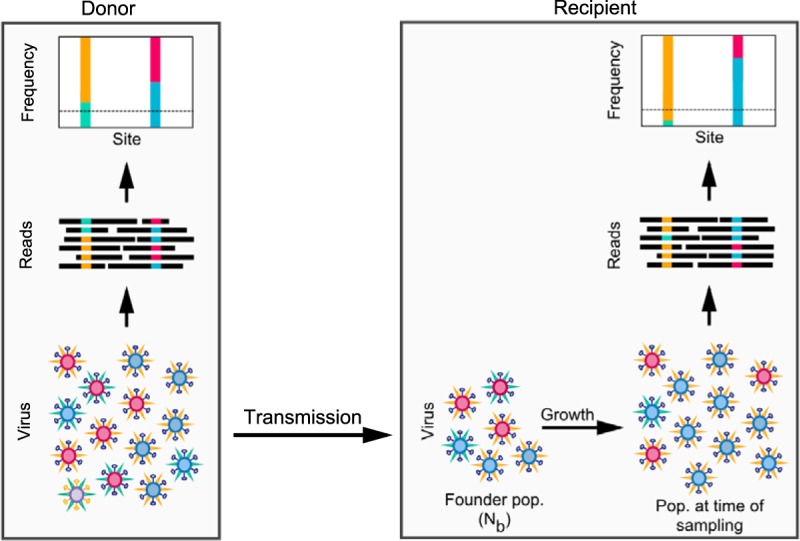
Schematic showing virus transmission from donor to recipient host. The number of virions that initiate infection in the recipient host is defined as the transmission bottleneck size or founding population size, *N_b_*. The viral sampling process is shown, with deep sequencing of the viral population resulting in reads that carry polymorphisms at certain nucleotide sites. The nucleotide readouts at any site can be used to estimate variant frequencies. Dashed horizontal lines in the variant frequency plots denote the variant calling cutoff or threshold. The goal is to estimate *N_b_* given data on variant frequencies in the donor and in the recipient, the total number of reads, and the number of variant reads at each of the variant sites identified in the donor.

Figure 1 provides a schematic of the data that are used for inferring transmission bottleneck sizes in the approaches that we consider in this study. Deep-sequencing data consist of short reads at various sites in the genome, obtained from both the infected donor and the recipient at, generally, a single time point for each individual. The short-read data are used to identify viral variants in the donor and recipient hosts. Comparison of these variants' frequencies across donor-recipient transmission pairs allows us to infer the transmission bottleneck size (*N_b_*), the number of virions comprising the founding viral population at the onset of infection in the recipient host. We specifically define *N_b_* as the number of virions that successfully establish lineages that persist to the sampling time point. There may, however, be additional virions that transiently replicate in the recipient host but quickly die out and are therefore not included in *N_b_*.

Given the extent of sequencing error in deep-sequencing data, there can be a high degree of noise in the short-read data and, thereby, in the extent of polymorphisms present at nucleotide sites. To limit the spurious identification of variants arising from sequencing noise, it is common practice to use criteria, such as a variant calling threshold, to validate identified variants (31). The variant calling threshold is the minimum frequency at which a variant can almost certainly be distinguished from background sequencing error. This threshold frequency may be chosen according to generally accepted error rates for a specific sequencing platform, error rates informed by a control run, or error rates based on the concordance of variant calls from replicate sequence runs. For the commonly used Illumina sequencing platforms, variant thresholds tend to fall in the range of 0.5 to 3% (24–26, 28, 32–35). Conservative variant calling cutoffs are often used, as they ensure that sequencing artifacts are excluded. However, conservative frequency cutoffs may have effects on transmission bottleneck size analyses due to variants that are not called in the recipient host despite being present. Such “false negatives” in the recipient have the potential to skew the inferred transmission bottleneck size toward inappropriately low values.

We present methods for inferring the transmission bottleneck size from deep-sequencing data, paying special attention to the effects of false-negative variant calls. We first introduce the beta-binomial sampling method that we have developed for bottleneck size inference, which further incorporates the effects of stochastic pathogen dynamics in recipient hosts. For comparison, we then summarize two existing methods of bottleneck size inference in the literature: the presence/absence method and the binomial sampling method. Of note, all three of these methods assume that the genetic diversity of the pathogen is entirely neutral, such that selection does not impact variant frequency dynamics. These methods further assume independence between variant sites. We address the limitations of these assumptions in the Discussion.

### Bottleneck size inference allowing for stochastic pathogen dynamics in the recipient host.

The beta-binomial sampling method for inferring the bottleneck size allows variant allele frequencies in the recipient host to change between the time of founding and the time of sampling (Fig. 1), as the result of stochastic pathogen replication dynamics early in infection. We consider two implementations of the beta-binomial sampling method: an approximate version that assumes an infinite read depth and an exact version that incorporates sampling noise arising from a finite number of reads. The derivation of the beta-binomial sampling method can be found in Materials and Methods.

In the approximate version, the likelihood of a transmission bottleneck size, *N_b_*, given variant frequency data at site *i*, is given by

(1) $$L(N_{b}{)}_{i}\;=\;\overset{N_{b}}{\underset{k\;=\;0}{\sum }}p\_beta(\nu_{R,i}|k,\;N_{b}\;-k)\;p\_bin(k|N_{b},\;\nu_{D,i})$$

where ν_*R*,*i*_ is the variant frequency at site *i* in the recipient and *p_beta*(ν_*R*,*i*_|*k*, *N_b_* − *k*) is given by the beta probability density function parameterized with shape parameters *k* and *N_b_* − *k* and evaluated at ν_*R*,*i*_. The term *p_bin*(*k*|*N_b_*, ν_*D*,*i*_) denotes the binomial distribution evaluated at *k* and parameterized with *N_b_* number of trials and a success probability of ν_*D*,*i*_, where ν_*D*,*i*_ is the variant frequency at site *i* in the donor. If the donor variant at site *i* is not detected in the recipient, this may be because it is truly absent from the recipient or because it falls below the variant calling threshold. To allow for both of these possibilities, the likelihood that the transmission bottleneck size is *N_b_*, given that the variant at site *i* was not detected, is given by

(2) $$L(N_{b}{)}_{i}\;=\;\overset{N_{b}}{\underset{k\;=\;0}{\sum }}\left[p\_beta\_cdf(\nu_{R,i}<\;T|k,\;N_{b}\;-\;k)\;p\_bin(k|N_{b},\;\nu_{D,i})\right]$$

where *T* is the variant calling threshold and *p_beta_cdf*(ν_*R*,*i*_ < *T*|*k*, *N_b_* − *k*) is given by the beta cumulative distribution function evaluated at the variant calling threshold.

In the exact version of the beta-binomial sampling method, we incorporate sampling error by modifying equations 1 and 2 to consider the number of variant reads and the number of total reads at variant site *i* in the recipient, *R_var_*_,*i*_ and *R_tot_*_,*i*_, respectively. The likelihood expression for the bottleneck size at site *i* becomes

(3) $$L(N_{b}{)}_{i}\;=\;\overset{N_{b}}{\underset{k\;=\;0}{\sum }}p\_betabin(R_{\mathrm{var},i}|R_{tot,i},\;k,\;N_{b}-k)\;p\_bin(k|N_{b},\;\nu_{D,i})$$

where *p_betabin*(*R_var_*_,*i*_|*R_tot_*_,*i*_, *k*, *N_b_−k*) is given by the beta-binomial probability density function evaluated at *R_var_*_,*i*_ and parameterized with *R_tot_*_,*i*_ number of trials and parameters *k* and *N_b_−k*. If the donor-identified variant at site *i* is not detected in the recipient, we again construct the likelihood that allows for this variant to be either absent from the recipient or below the variant calling threshold:

(4) $$L(N_{b}{)}_{i}\;=\;\overset{N_{b}}{\underset{k\;=\;0}{\sum }}p\_betabin\_cdf(R_{\mathrm{var},i}\;<\;TR_{tot,i}|R_{tot,i},k,\;N_{b}-k)\;p\_bin(k|N_{b},\;\nu_{D,i})$$

where, in this case, *p_betabin_cdf*(*R_var_*_,*i*_ < *TR_tot_*_,*i*_|*R_tot,i_*, *k*, *N_b_−k*) is given by the beta-binomial cumulative distribution function evaluated at the number of reads that would qualify as falling at the variant calling threshold.

We expect that the maximum likelihood estimate (MLE) of *N_b_* inferred with the approximate method will converge to the MLE of *N_b_* inferred with the exact method when the read coverage is high. The benefit of using the approximate version, when appropriate, is that the incorporation of sampling error is computationally intensive.

Once transmission bottleneck sizes have been estimated by using either the approximate or exact beta-binomial sampling method, the probability that a variant is truly present/absent in the recipient and the probability that a variant is simply called present/absent in the recipient (under the assumption of infinite coverage) can be determined for any given donor variant frequency.

### Existing methods for inferring transmission bottleneck sizes. (i) Presence/absence method of bottleneck size inference.

The simplest approach to estimating transmission bottleneck sizes from pathogen deep-sequencing data is to calculate variant frequencies in donor hosts and then use information on the presence/absence of these variants in recipient hosts to quantify the bottleneck size. Studies that have adopted this approach have been reported previously (9, 36). Given a variant, *i*, present at frequency ν_*D*,*i*_ in the donor and a founding population size of *N_b_*, the probability that the variant was not transferred to the recipient is simply given by (1 − *ν_D,i_*)*^N^_b_* (9, 36). Correspondingly, the probability that at least one virion in the founding population carried the variant allele is given by 1 − (1 − *ν_D,i_*)*^N^_b_*. From these expressions, the likelihood of the founding population size of *N_b_* in a donor-recipient pair is simply calculated by multiplying the probabilities of the observed outcomes across the variant sites:

(5) $$L(N_{b})\;=\;\overset{V_{absent}}{\underset{j\;=\;1}{\prod }}(1\;-\;\nu_{D,j}{)}^{N_{b}}\;\overset{V_{present}}{\underset{k\;=\;1}{\prod }}\left[1\;-\;(1\;-\;\nu_{D,k}{)}^{N_{b}}\right]$$

where *j* indexes the viral variants that are absent in the recipient, *k* indexes the viral variants that are present in the recipient, *V_absent_* is the total number of variants that are called absent in the recipient, and *V_present_* is the total number of variants that are called present in the recipient. The total number of variants identified in the donor is given by *V_absent_* + *V_present_*.

The presence/absence method considers only the detection of donor-identified variants in the recipient host and, therefore, is especially prone to the effects of false-negative variants. Moreover, accounting for the variant calling threshold to ameliorate these effects is not possible with this method. Due to the inability of this method to account for false negatives, we expect that the transmission bottleneck estimates inferred with the presence/absence method will be considerably lower than the bottleneck size estimates inferred by the beta-binomial sampling method.

### (ii) Binomial sampling method of bottleneck size inference.

The second approach, or class of approaches, from the literature for inferring transmission bottleneck sizes is based on a binomial sampling process. Studies that have adopted this general kind of approach have been reported previously (28, 30). We describe a version of this approach that parallels the beta-binomial sampling method that we describe above. The binomial sampling approach makes use of donor-identified variant frequencies in the donor and both the number of variant reads and the number of total reads in the recipient, at each donor-identified variant site. The likelihood expression for the bottleneck size, given these data at site *i*, is given by

(6) $$L(N_{b}{)}_{i}\;=\;\overset{N_{b}}{\underset{k\;=\;0}{\sum }}p\_bin(R_{\mathrm{var},i}|R_{tot,i},\;\frac{k}{N_{b}})\;p\_bin(k|N_{b},\;\nu_{D,i})$$

where *p_bin*(*R_var_*_,*i*_|*R_tot_*_,*i*_, *k*/*N_b_*) is given by the binomial probability density function evaluated at *R_var_*_,*i*_. The term *p_bin*(*k*|*N_b_*, ν_*D*,*i*_) is again given by the binomial distribution. For variants called as absent in the recipient host, the likelihood of the transmission bottleneck size is given as

(7) $$L(N_{b}{)}_{i}\;=\;\overset{N_{b}}{\underset{k\;=\;0}{\sum }}p\_bin\_cdf\;(R_{\mathrm{var},i}\;<\;TR_{tot,i}\;|R_{tot,i},\frac{k}{N_{b}})\;p\_bin(k|N_{b},\;\nu_{D,i})$$

where *p_bin_cdf* is the binomial cumulative distribution function. The derivation of the binomial sampling method can be found in Materials and Methods.

The sole difference between the beta-binomial sampling method and the binomial sampling method is that the binomial sampling method does not account for stochastic dynamics of the pathogen early on in the recipient. These stochastic dynamics enable the frequencies of variants in a recipient at the time of sampling to differ from those at the time of founding (Fig. 1). Because the binomial sampling method does not incorporate this source of frequency variation, we expect there to be smaller frequency deviations between variants in donor-recipient pairs under the assumption of a single-generation binomial sampling model than in a model that allows for these stochastic dynamics, for a given bottleneck size. To explain a given pattern of donor-recipient frequency pairs, *N_b_* estimates are thus expected to be significantly lower for the binomial sampling method than for the beta-binomial sampling method. Application of the binomial sampling method will therefore yield a conservative (lower-bound) estimate of *N_b_*, as previously remarked upon (30).

## RESULTS

### Results on simulated data.

**FIG 2:**
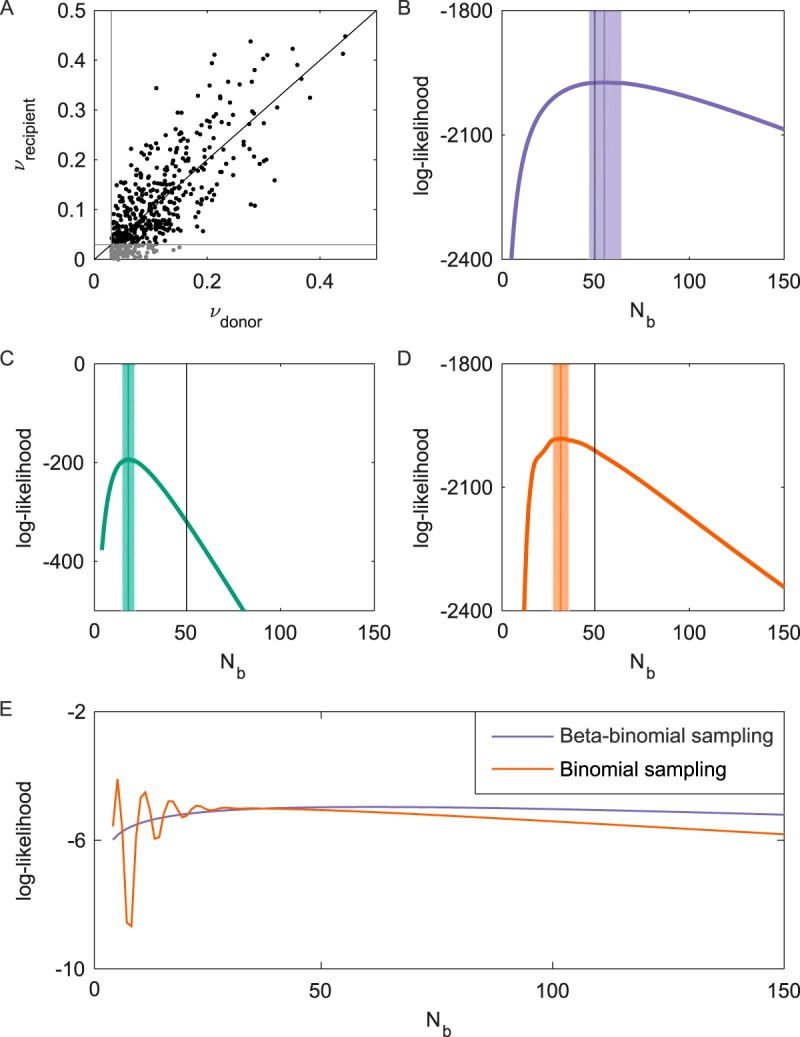
Estimated transmission bottleneck sizes for a simulated NGS data set. (A) Scatterplot showing the frequencies of donor-identified variants against the corresponding frequencies of these variants in the recipient. Points in black are variants that are called present in the recipient host. Points in gray are variants that are called absent in the recipient host. The black line shows where ν_donor_ equals ν_recipient_. Gray lines show the variant calling threshold of 3%. (B) Log-likelihood curve for the beta-binomial sampling method over a range of *N_b_* values. The MLE is 55 virions (95% CI = 47 to 64 virions). The likelihood at MLE equals −1,972.7. (C) Log-likelihood curve for the presence/absence method over a range of *N_b_* values. The MLE equals 19 virions (95% CI = 16 to 22 virions). (D) Log-likelihood curve for the binomial sampling method over a range of *N_b_* values. The MLE equals 32 virions (95% CI = 28 to 36 virions). The likelihood at MLE equals −1,981.8. In panels B to D, vertical black lines show the true transmission bottleneck size, *N_b_*, of 50. Vertical colored lines show the MLEs, and shaded areas show the 95% confidence intervals, determined by using the likelihood ratio test. (E) Likelihood surfaces for a single variant present in the recipient at a frequency of 16.9% under the beta-binomial sampling model and the binomial sampling model.

To examine the abilities of the three methods described above to accurately infer transmission bottleneck sizes, we used a simulated data set of one donor-recipient pair (see Materials and Methods). The data set was generated under the assumption of stochastic pathogen dynamics in the recipient host between the time of infection and the time of sampling. While this assumption matches the assumption for the beta-binomial sampling method, we feel that it is also biologically the most realistic assumption. In this data set, 109 out of the 500 donor-identified simulated variants were called absent in the recipient host (Fig. 2A). The majority of these variants were present in the recipient host but below our variant calling threshold of 3% and therefore were false negatives. The beta-binomial sampling method, as expected, recovers the true bottleneck size of 50 virions (Fig. 2B). In contrast, both the presence/absence method (Fig. 2C) and the binomial sampling method (Fig. 2D) significantly underestimate the simulated bottleneck size. The underlying reasons for these methods' inability to recover the true bottleneck size differ. For the presence/absence method, this underestimation can be attributed to false-negative variant calls. For the binomial sampling method, we were able to statistically account for the variant calling threshold effects; the underestimation of this method, therefore, is attributed solely to this method not accounting for stochastic pathogen dynamics in the recipient. The binomial sampling method instead assumes deterministic viral growth from the time of founding to the time of sampling (see Materials and Methods). Because more sampling stochasticity is present at smaller bottleneck sizes, the binomial sampling method underestimates the simulated bottleneck size in its attempt to reproduce the observed variation in variant frequencies by inappropriately constricting *N_b_*.

Given that the binomial sampling model and the beta-binomial model were fit to the same data, the relative performances of these models can be assessed by using model selection approaches. The maximum likelihood obtained by using the beta-binomial sampling method was significantly higher than the maximum likelihood obtained by using the binomial sampling method (Fig. 2B and D), indicating that the beta-binomial sampling model is statistically preferred over the binomial sampling model. We can further take into consideration the smoothness of the likelihood curves in our choice of model, with multimodal/rugged likelihood curves being undesirable outcomes. In Fig. 2E, we plot the likelihood curves for one variant under the likelihood expression of the beta-binomial sampling method and under the expression of the binomial sampling method. The rugged likelihood surface of the binomial sampling model arises because of this method's stringent assumption that variant frequencies remain fixed between the time of infection of the recipient and the time of sampling. In contrast, the beta-binomial sampling method allows for stochastic changes in variant frequencies during viral growth, relaxing the assumption that the viral population at the time of sampling needs to perfectly reflect the founding viral population. As a result, likelihood curves of the beta-binomial sampling model do not show large differences in likelihood values for small differences in *N_b_*, further indicating that the beta-binomial sampling model is preferable.

**FIG 3:**
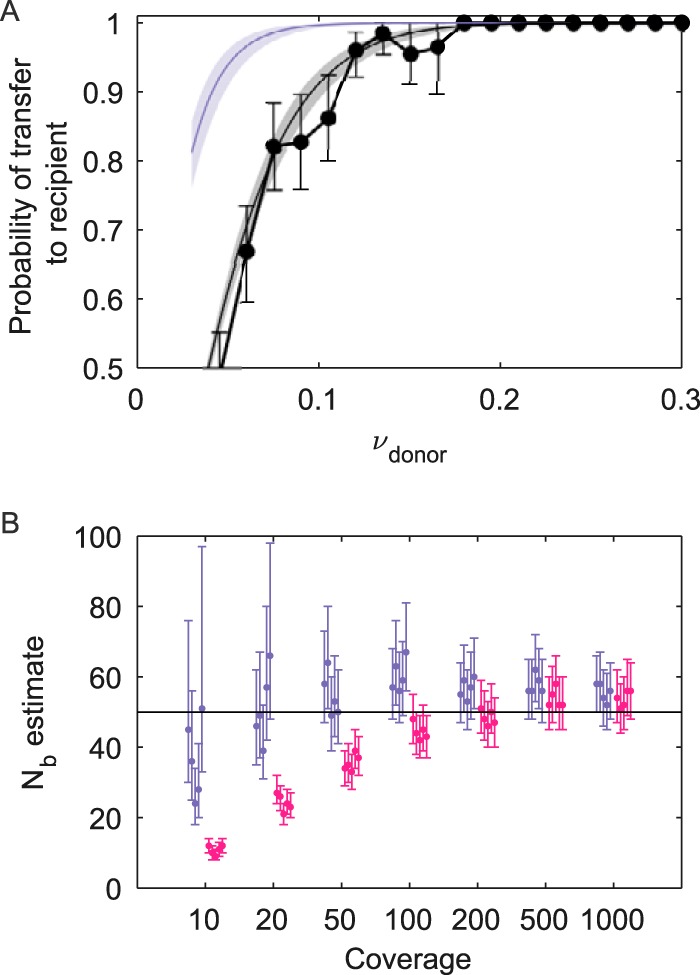
Additional results from application of the beta-binomial sampling method to the simulated data set. (A) Probability of a donor-identified variant being either transferred or observed as transferred (“called”) in a recipient host, as a function of donor variant frequencies. The observed probabilities of donor-identified variants being called in a recipient host are shown in black, calculated directly from the simulated data set using 3% frequency bins. The 95% confidence intervals assume that the probability of variant transfer follows a binomial distribution with the number of trials being the number of donor-identified variants present in a frequency bin and the success probability given by the calculated probability of transferred variants observed in the frequency bin. Probabilities of donor-identified variants being truly present in a recipient host are shown in purple, given bottleneck size estimates from the beta-binomial sampling method. Probabilities of donor-identified variants being called present in a recipient host are shown in gray, given bottleneck size estimates from the beta-binomial sampling method. (B) *N_b_* estimates for simulated data sets that differ in coverage levels. At each coverage level, 5 data sets were generated under the same parameters and assumptions as those for the data set shown in Fig. 2A. Both the exact beta-binomial sampling method and the approximate version of this method were used to estimate *N_b_* for each data set. *N_b_* maximum likelihood estimates and 95% confidence intervals are shown in purple for the exact beta-binomial sampling method and in pink for the approximate method.

Given an estimate of the transmission bottleneck size, the probability that a variant is transferred to a recipient host can be calculated by using the expression 1 − (1 − *ν_D,i_*)*^N^_b_*, where *ν_D,i_* is the frequency of variant *i* present in the donor host and *N_b_* is the bottleneck size estimate. In Fig. 3A, we plot this probability of variant transfer over a range of donor variant frequencies for the simulated data set. In this figure, we further plot “observed” probabilities of variant transfer, using a variant calling threshold of 3% for the simulated data set. Finally, in Fig. 3A, we plot the observed probabilities of variant transfer as predicted under the beta-binomial sampling method, evaluated at the transmission bottleneck size estimated. We see first that the true probabilities of variant transfer greatly exceed those that are observed in the data set given the variant calling threshold of 3%. However, this method's calculated predictions of observed variant transfer probabilities fall within the 95% confidence intervals (CIs) for the probabilities of variant transfer observed in the data set.

As described in the introduction, the exact beta-binomial sampling method that we developed accounts for sampling noise arising from finite read coverage. If we ignore sampling noise, we can estimate bottleneck sizes more rapidly using the approximate method, described by equations 1 and 2. In Fig. 3B, we show bottleneck size estimates over a range of different coverage levels for both the exact and approximate beta-binomial sampling methods. At high coverage levels (>200 reads), both implementations of the beta-binomial sampling method yield similar bottleneck size estimates and are able to recover the simulated bottleneck size of 50 virions. For lower levels of coverage, however, this approximation starts to fail and will lead to a considerable underestimation of *N_b_*, indicating that the approximate beta-binomial sampling method is inappropriate for low coverage levels. We also note that even at high coverage levels, a slight overestimation of the bottleneck size is apparent for both the beta-binomial and the approximate beta-binomial sampling methods. This overestimation can be attributed to the rare false-positive identification of variants in the recipient (instances of a variant that is absent in the recipient being called present) and, more generally, a slight inflation of variant frequencies with sequencing error. Overestimation no longer occurs when these methods are applied to data sets that are simulated in the absence of sequence error (results not shown).

### Transmission bottleneck size estimation for human influenza A virus.

**FIG 4:**
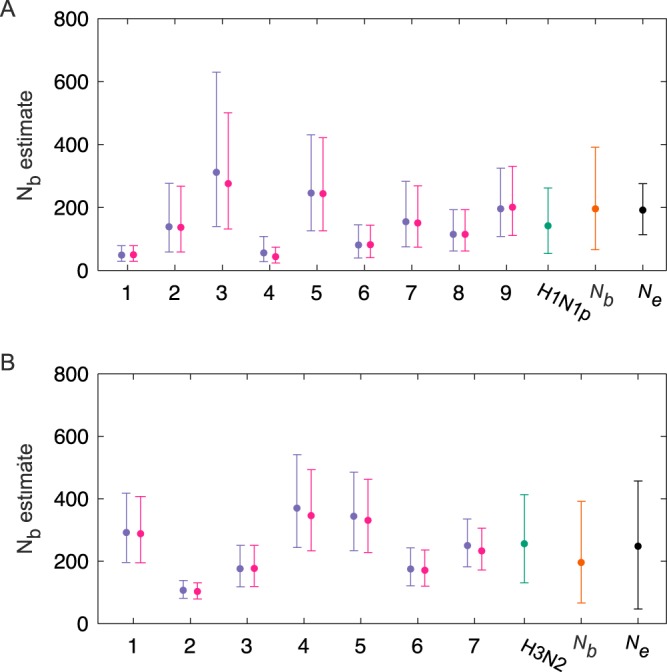
Transmission bottleneck sizes estimated for influenza A virus H1N1p (A) and H3N2 (B) transmission pairs. *N_b_* estimates are shown for the exact beta-binomial sampling method (purple) and the approximate version of this method (pink). Bars show means and 95% CIs, calculated by using the likelihood ratio test. Overall transmission bottleneck sizes estimated across H1N1p transmission pairs (“H1N1p”) (teal), across H3N2 transmission pairs (“H3N2”) (teal), and across both subtypes (“*N_b_*”) (orange), under the assumption of a negative binomial distribution, are also shown. Previous estimates by Poon et al. (28) are also shown (“*N_e_*”) for H1N1p and H3N2 (black). Bars for the estimates by Poon et al. show mean estimated effective population sizes and mean standard deviation ranges.

We first applied the beta-binomial sampling method for inferring transmission bottleneck sizes to the influenza A/H1N1p virus transmission pairs identified in an influenza virus NGS data set described in detail previously (28). We point the reader to this previous report for details on the data set, including coverage levels and how transmission pairs were inferred, etc. Poon et al. (28) estimated the mean effective population size for all H1N1p transmission pairs, *N_e_*, to be equal to 192 virions (mean standard deviation range, 114 to 276 virions). This approach considered the combined set of variants that were present at frequencies of ≥1% and that were shared by 8 identified household donor-recipient pairs (a total of 26 variants). In contrast to that analysis, we estimated transmission bottleneck sizes for each of the 9 transmission pairs separately, using a minimum variant frequency cutoff of 3% to call variants. We used a 3% cutoff based on concordance results from replicate sequencing runs, as described previously (28). The less conservative 1% cutoff used by Poon et al. (28) to estimate the effective population size was chosen to allow for more sites to be included in their analysis. Our analysis, using a total of 289 variants, estimated MLE bottleneck sizes ranging from 49 to 276 virions across the H1N1p transmission pairs (Fig. 4A). The bottleneck sizes inferred by the approximate beta-binomial sampling method did not differ significantly from those inferred by the exact method for any of the transmission pairs. This was expected, given high coverage levels across variant sites.

To summarize our results for the bottleneck size estimates for the H1N1p transmission pairs, we estimated parameters of a negative binomial distribution using all of the variant frequencies across the transmission pairs (see Materials and Methods). This negative binomial distribution was chosen because our results shown in Fig. 4A indicated that the variance in transmission bottleneck sizes is likely to exceed the mean. We further fit a Poisson distribution to the same data, and the negative binomial distribution was statistically preferred over the Poisson distribution using the Akaike information criterion (AIC) indicating that while a single infection may be initiated by a Poisson-distributed number of virions, different infections are likely to be initiated by founding population sizes that vary in their means. The MLE values for the negative binomial distribution's parameters were an *r* value of 5 and a *p* value of 0.966, resulting in a mean H1N1p transmission bottleneck size, *N_b_*, of 142 and a 95% range of 54 to 262 virions (Fig. 4A). While our overall bottleneck size estimates were consistent with the estimates of Poon et al. using a much more limited number of variants, our analysis further shows that the transmission bottleneck sizes varied considerably between transmission pairs.

We next used the beta-binomial sampling method to infer the transmission bottleneck sizes for each of the H3N2 transmission pairs of the influenza virus NGS data set. Poon et al. estimated the mean effective population size, *N_e_*, for H3N2 to be 248 virions (mean standard deviation range of 45 to 457 virions), again using a combined set of variants that were present at frequencies of ≥1% and that were shared by 6 identified household donor-recipient pairs (a total of 81 variants). Our analysis, considering each of the 7 identified H3N2 transmission pairs separately, inferred MLE bottleneck sizes ranging from 107 to 370 virions across the transmission pairs, using a total of 621 variants (Fig. 4B). Again, as expected, the *N_b_* sizes inferred by the approximate beta-binomial sampling method did not differ significantly from those inferred by using the exact beta-binomial sampling method. We again fit a negative binomial distribution to all of the variants across the transmission pairs and estimated MLE parameters of an *r* value of 9 and a *p* value of 0.966, resulting in a mean H3N2 transmission bottleneck size, *N_b_*, of 256 virions and a 95% range of 131 to 413 virions (Fig. 4B). We again observed that the overall bottleneck size estimate for H3N2 was consistent with the estimate by Poon et al., although the bottleneck size estimates varied considerably between transmission pairs.

### Overall influenza A virus transmission bottleneck sizes.

**FIG 5:**
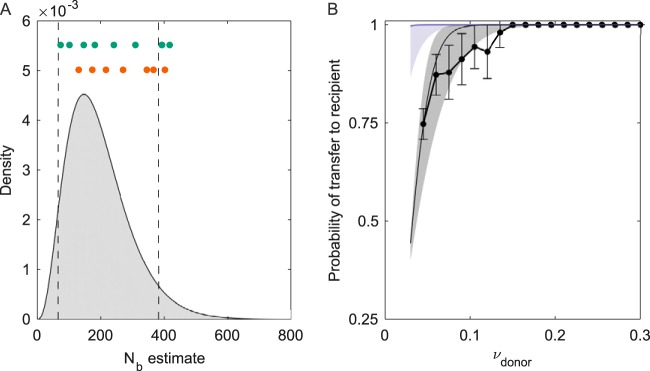
Overall influenza A virus bottleneck size estimates and probabilities of variant transfer under these estimates. (A) The negative binomial probability density function (pdf) describing overall transmission bottleneck sizes across H1N1p and H3N2 viral subtypes, parameterized with MLE values of an *r* value of 4 and a *p* value of 0.980. Vertical black lines show the 95% range of this distribution. The MLE bottleneck size estimates for the H3N2 (orange) and H1N1 (green) transmission pairs are shown above the pdf. (B) Probability of a donor-identified variant being either transferred or identified (called) in the recipient host as a function of donor variant frequency. Probabilities of a donor variant being present in a recipient host are shown in purple, given bottleneck size estimates provided by the negative binomial distribution shown in panel A. Probabilities of donor-identified variants being called present in a recipient host, given these same bottleneck size estimates and the assumptions of the beta-binomial sampling models, are shown in gray. The empirical probabilities of donor-identified variants being called in a recipient, as calculated from the combined H1N1p and H3N2 data sets over 3% frequency bins, are shown in black.

We next sought to determine whether influenza A/H1N1p and influenza A/H3N2 virus subtypes statistically differed from one another in bottleneck sizes. We found that the H1N1p and H3N2 distributions of transmission bottleneck size MLEs did not differ significantly from one another (*P* = 0.15 using the Kolmogorov-Smirnov test). Given this finding, we fit a negative binomial distribution to all of the variants across the data sets of both subtypes, arriving at MLE parameters of an *r* value of 4 and a *p* value of 0.980 for the parameters of the negative binomial distribution. These parameters correspond to a mean bottleneck size, *N_b_*, of 196 virions and a 95% range of 66 to 382 virions (Fig. 4A and B). We show the probability density function for this negative binomial distribution in Fig. 5A. We further plot the expected probability of variant transfer for this bottleneck size estimate (Fig. 5B), similar to what we show for the simulated data set in Fig. 3A. Finally, we plot the probability of observed variant transfer under this *N_b_* estimate, under the assumptions of the beta-binomial sampling model. The agreement between the probability of observed variant transfer and the empirical data indicates that variant calling thresholds again make it appear that variant transfer from donor to recipient is much less likely than it is, given bottleneck size estimates based on variant frequencies.

### Relationship between donor temperature and estimated bottleneck size.

Given the extent of variation in bottleneck size estimates across transmission pairs, we next considered whether certain characteristics of the donor may account for some of the observed variation. Available metadata for donor individuals included demographic data (age and gender), 2009 vaccination status, oseltamivir treatment, temperature measurements, and symptom scores (available at http://web.hku.hk/~bcowling/influenza/HK_H1N1_study.htm). Symptom scores were calculated as the number of symptoms present at the time of measurement, with considered symptoms being headache, sore throat, cough, myalgia, runny nose, and phlegm. As possible explanatory variables, we limited our analysis to temperature measurements and symptom scores. This is because, with the exception of antiviral treatment, a clear hypothesis relating any of these metadata variables to inferred transmission bottleneck sizes was lacking. We did not consider antiviral treatment as a possible explanatory variable because the time at which the antiviral was administered relative to the time of transmission was unknown.

**FIG 6:**
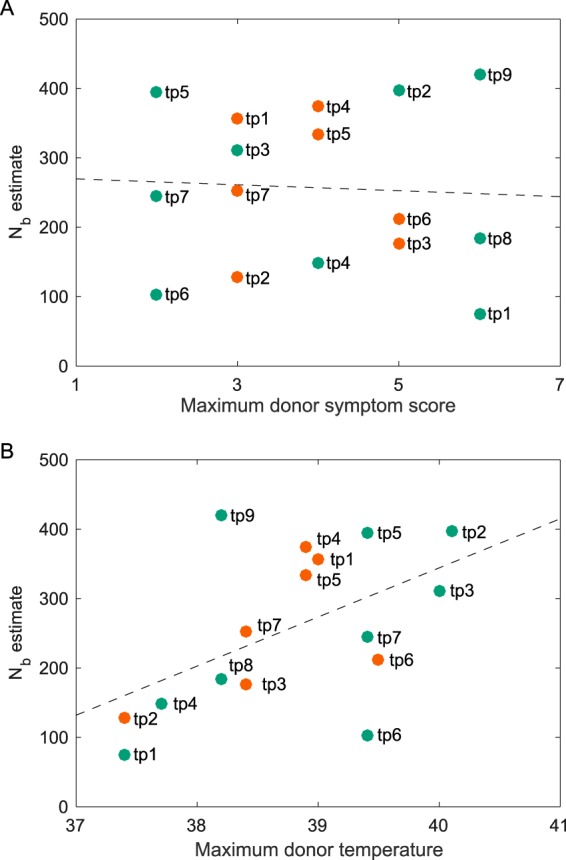
Relationships between transmission bottleneck size estimates and characteristics of the donor host. (A) Relationship between inferred transmission bottleneck sizes and the donor's maximum symptom score. (B) Relationship between inferred transmission bottleneck sizes and the donor's maximum temperature. In panels A and B, points are labeled by transmission pair, with green points denoting H1N1p transmission pairs and orange points denoting H3N2 transmission pairs. Dashed lines show marginal linear regressions calculated from the multiple linear regression using both maximum donor temperature and maximum donor symptom score as predictor variables. Maximum donor temperature was found to be a significantly positive predictor of *N_b_* (*r* = 0.79; *P* = 0.035), while maximum donor symptoms were not predictive of *N_b_* (*r* = 0.13; *P* = 0.52).

We determined the relationship between inferred bottleneck sizes and both donor temperature and symptoms using multiple-linear-regression analysis. Specifically, we used the maximum donor temperature and the maximum symptom score as predictors of the inferred bottleneck size. The results of this regression indicated that donor symptoms were not a significant predictor of inferred transmission bottleneck sizes (*P* = 0.52) (Fig. 6A). However, donor temperature was found to be a significant predictor of inferred bottleneck sizes (*P* = 0.035) (Fig. 6B) at a significance level of an α value of 0.05, with higher donor temperatures being positively associated with larger transmission bottleneck sizes. Using a highly conservative significance level of an α value of 0.025, determined by applying the Bonferroni correction for multiple comparisons, this *P* value falls slightly above the level of significance. We note, however, that the Bonferroni correction applied to results of multiple-linear-regression analyses has been shown to be overly conservative (37).

## DISCUSSION

Here, we have introduced a new method for estimating the transmission bottleneck size of pathogens from next-generation sequencing data from donor-recipient pairs. We have further analyzed how well this beta-binomial sampling method performs in comparison to two existing methods in the literature: the presence/absence method and the binomial sampling method. Using a simulated data set, we have demonstrated that both the presence/absence method and the binomial sampling method (for different reasons) systematically underestimate the transmission bottleneck size and that the latter can lead to undesirable rugged likelihood curves. In contrast, the beta-binomial sampling method, as expected, is able to recover the simulated bottleneck size (Fig. 2B) and is able to accurately predict the probability that a donor variant would be identified in a recipient host under a given variant calling threshold (Fig. 3A). Application of the beta-binomial sampling method to a previously reported H1N1p and H3N2 NGS data set showed a high degree of heterogeneity between bottleneck size estimates across transmission pairs (Fig. 4). A negative binomial distribution was fit to all of the variants, yielding an overall mean *N_b_* of 196 virions and a 95% range of 66 to 382 virions (Fig. 4A and B and 5A).

The bottleneck sizes that we estimated for the H1N1p and H3N2 transmission pairs are close to previous estimates of the effective population size, *N*_e_, arrived at by Poon et al. for this data set (28), although we were able to further estimate transmission bottleneck sizes by transmission pair, and our method was able to make use of a much larger number of identified variants. Our bottleneck size estimates are consistent with the more qualitative observations of loose transmission bottlenecks for influenza A virus transmission in horses (20, 22, 23), pigs (20, 21), and dogs (19). Our *N_b_* estimates, however, are considerably larger than the previous bottleneck sizes estimated for this virus by Varble et al. (27), Frise et al. (29), and McCaw et al. (17). The experimental study by Varble et al. showed that the route of transmission affected the bottleneck size, with contact transmission giving rise to larger bottlenecks. Those researchers found that, of the 71 to 100 distinct viral tags, only 7 to 24 of these tagged viruses were detected in the recipients following infection via direct contact (27). The number of distinct viral tags, however, might reflect the lower limit of the bottleneck size because it is possible that more than one virion passing through the bottleneck would have the same tag. Frise et al. reported a mean bottleneck size of 28.2 infectious genomes for contact transmission of an efficiently transmitted H1N1 strain in ferrets, although they were unable to identify an upper limit to the bottleneck size confidence interval (29). Both of those estimates are much larger than the earlier estimate of 3.8 virions by McCaw et al. for contact transmission of H1N1 in ferrets (17). While there are other studies that have estimated the transmission bottleneck size in the context of viral adaptation to a new host species (24–26), comparisons with those studies are inappropriate because these bottlenecks are subject to strong selective forces, which considerably narrow the transmission bottleneck size (38).

The *N_b_* estimates for influenza virus transmission in the data set described here, in both our study and the original analysis by Poon et al., are considerably higher than previous quantitative estimates of the bottleneck size for contact transmission of IAV (17, 27, 29). Notably, those previous estimates of *N_b_* were arrived at by using data from experimental ferret infections. With a recent analysis showing that secondary attack rates in ferret studies are considerably higher than human secondary attack rates, controlling for infecting subtype (39), one possibility for these discrepancies is that ferrets and other small mammals may require fewer influenza A virions to successfully initiate infection.

In particular, the bottleneck size estimate by McCaw et al. was significantly lower than our *N_b_* estimates for contact transmission of influenza virus (17). One possible explanation for the low *N_b_* estimate is that the “competitive-mixture” method that those authors used to calculate bottleneck size considers only two viral populations, analogous to the estimates derived from a single variant in the methods that we considered. The competitive-mixture method is thus highly susceptible to fluctuations between donor and recipient variant frequencies arising from stochastic viral dynamics in the recipient. Thus, for the same reason that the binomial sampling method that we describe here underestimates bottleneck sizes, we would expect this competitive-mixture method to considerably underestimate bottleneck sizes. However, this method is free of one of the necessary assumptions made for each the three methods that we considered, namely, that the variants considered are independent. The independence assumption is clearly violated in this data set given the extensive genetic linkage within influenza virus gene segments (40). We can, however, somewhat control for the effects of linkage by selecting only one variant per gene segment. This data-thinning approach still assumes independence across gene segments that, while not ideal, may be supported by recent experimental evidence showing high levels of reassortment *in vitro* (41). If intrahost reassortment occurs at similar rates *in vivo*, then sampling of only one variant per gene segment should remove much of the bias due to linkage.

The methods that we considered make other assumptions that may also have impacted transmission bottleneck size estimates. These assumptions include that (i) donor-identified variants did not originate *de novo* in any recipient hosts, (ii) variants were biallelic, and (iii) variants were selectively neutral. Significant levels of *de novo* evolution of variants in recipient hosts would artificially increase estimated bottleneck sizes. Therefore, these methods may not be appropriate for pathogens causing chronic infections, such as HIV, where sampling of the recipient host can occur years after the initiation of infection. However, we do not expect substantial *de novo* evolution of variants to occur over the course of an acute influenza virus infection based on recent findings (38) and the observation that the vast majority of recipient-identified variants were also present in the donor. Therefore, we do not expect this assumption to have significantly influenced our bottleneck size estimates for influenza virus.

We also do not expect the second assumption—that loci are biallelic—to have biased our bottleneck size estimates. This is because no sites used in our bottleneck size calculations contained more than one variant allele above our variant calling threshold of 3%. This assumption, however, could be removed in future uses of the beta-binomial sampling method by appropriately modifying the likelihood expressions to account for more than one variant per site.

The third assumption, of selective neutrality, is the one that could greatly affect the accuracy of our bottleneck size estimates if not met. Selection, either for or against a variant, would lead to larger differences in variant frequencies between a donor and a recipient host than would be expected for neutral variants. Larger differences in variant frequencies would bias the estimated transmission bottleneck sizes toward smaller values. Thus, our bottleneck size estimates, which assume neutrality, are necessarily conservative estimates.

In addition to confirming the large transmission bottleneck size for IAV in this data set, we have shown that estimated bottleneck sizes vary considerably across transmission pairs. This observation is in agreement with data from previous influenza virus transmission studies in ferrets. Those studies showed that the bottleneck stringency for IAV is greatly influenced by the route of transmission, with contact transmission being much looser than respiratory/airborne transmission (27, 29). Our method's ability to infer bottleneck sizes of individual transmission pairs means that such analyses could potentially distinguish the route of transmission. Moreover, our analysis identified an association between the severity of infection of the donor, as measured by temperature, and the size of the transmission bottleneck, where more severe infections were associated with larger bottlenecks. This finding is intriguing, given that a previous study showed a positive relationship between host temperature and viral load during early infection (42), and another study showed a positive relationship between host temperature and nasal shedding (43). Our finding suggests that donor viral load and/or nasal shedding levels may impact transmission bottleneck sizes. Our finding that donor symptom scores do not explain any variation in bottleneck size estimates across transmission pairs is perhaps not surprising, given that some of the symptoms included in the score (e.g., headache) are unlikely to contribute to donor infectiousness.

In this study, we have developed a new statistical approach that can be used to accurately infer transmission bottleneck sizes for acute viral infections, such as influenza virus, respiratory syncytial virus (RSV), and norovirus, using NGS data from identified donor-recipient pairs. This beta-binomial sampling method accounts for the possibility of false-negative variants that are not called as present due to necessary variant calling thresholds. This method further accounts for changes in variant frequencies between the time of infection of the recipient and the time of pathogen sampling from the recipient that arise due to stochastic replication dynamics early in infection. Given the importance of the transmission bottleneck size in regulating the rate of pathogen evolution at the level of the host population, estimation of the transmission bottleneck size is a necessary component in the analysis of pathogens important to public health. Although methods such as viral tagging to estimate the bottleneck size for experimental infections exist, these techniques are not applicable to natural infections. Hence, this work provides a strong foundation for future estimations of bottleneck sizes from viral sequence data that, importantly, can be applied to clinical samples.

## MATERIALS AND METHODS

### Development of the beta-binomial sampling method.

Here, we derive the beta-binomial sampling method for inferring transmission bottleneck sizes from pathogen NGS data. The final likelihood expressions for this method are provided in equations 3 and 4. As described above, this method allows variant frequencies in the recipient host to change between infection and sampling (Fig. 1) due to stochastic pathogen dynamics occurring during the process of replication. More concretely, early in infection, when there are only a small number of replicating virions, stochasticity in viral growth is expected to have a large effect. For a stochastic birth-death process with a constant birth rate, λ, and a constant death rate, μ, the probability mass function for the viral population size originating from a single virion that successfully establishes infection (44) is given by

(8) $$P\;(N_{k}\;(t)\;=\;k)\;=\;(1\;-\;\eta_{t})\;{\eta_{t}}^{k\;-\;1},\;k\;\geq \;1$$

where *t* is the time of sampling and $\eta_{t}\;=\;\frac{\lambda \;(e^{(\lambda \;-\;\mu {)}^{t}\;-1})}{{\lambda e}^{(\lambda -\mu {)}^{t}}-\;\mu }$. For the bursty replication that characterizes many viruses, equation 8 is still approximately true at long times with an adjusted value of η*_t_*.

The population sizes stemming from each of the *N_b_* founding virions, contingent on their successful establishment, are thus geometrically distributed random variables. As these population sizes are likely to be very large at the time of sampling, we can approximate them as being exponentially distributed random variables. Under this approximation, the distribution of the fractions of the population that descend from each of the founding virions is Dirichlet(1,1,…1), with *N_b_* 1's, one for each ancestor. A subset, *k*, of these founder virions carries the variant allele; the remaining subset of these founder virions (*N_b_* − *k*) carries the reference allele. Collapsing the Dirichlet distribution yields that the fraction of the population carrying the variant allele is distributed as Beta(*k*, *N_b_* − *k*). Remarkably, this fraction does not depend on the within-host viral birth rate, λ; the death rate, μ; the time of sampling, *t*; or the burstiness of replication. To obtain the overall likelihood of population bottleneck size, *N_b_*, we simply have to consider all possible scenarios of how many virions out of the total *N_b_* virions transferred carried the variant allele. Under the assumption that the founding pathogen population is randomly sampled from the pathogen population of the donor host, the probability that the founding population of *N_b_* virions carries *k* variant alleles is given by the binomial distribution $p\_bin\;(k|N_{b},\;v_{D,i})\;\equiv \;\mathrm{Pr}\;(X\;=k|N_{b},\;v_{D,i})\;=\left(\underset{k}{N_{b}}\right)\;(v_{D,i}{)}^{k}\;(1\;-\;v_{D,i}{)}^{N_{b}\;-\;k}$, where the number of trials is given by *N_b_* and the success probability is given by ν_*D*,*i*_, the frequency of variant *i* in the donor. Thus, the overall likelihood of population bottleneck size, *N_b_*, for variant *i* is given by equation 1, where ν_*R*,*i*_ is the frequency of variant *i* in the recipient and the term *p_beta*(ν_*R*,*i*_|*k*, *N_b_* − *k*) is given by the beta probability density function, evaluated at ν_*R*,*i*_.

Accommodating sampling noise arising from a finite number of reads is simple, leading to minor modifications to the above-described equation (equation 1), resulting in equation 3, where *R_var_*_,*i*_ is the number of reads of the variant allele in the recipient sample at site *i* and *R_tot_* is the total number of reads at that site. The term *p_betabin*(*R_var_*_,*i*_|*R_tot_*_,*i*_, *k*, *N_b_−k*) is given by the beta-binomial distribution evaluated at *R_var_*_,*i*_ and parameterized with *R_tot_*_,*i*_ as the number of trials and parameters *k* and *N_b_*. Equation 3 thus incorporates noise both from the sampling process itself and from the process of stochastic pathogen growth. The overall likelihood of bottleneck size *N_b_* for a transmission pair is simply the product of the site-specific likelihoods.

As mentioned above, we expect that variant calling thresholds will impact the likelihood calculations used in the bottleneck size estimation. These thresholds will force some variant alleles in the recipient viral population to be called absent when they are actually present at frequencies below the value of the chosen threshold. Since a true absence of a variant allele is more likely at smaller bottleneck sizes, conservative variant calling thresholds will bias *N_b_* estimates toward lower values. Simply excluding variants that are called absent from the analysis, however, will also bias bottleneck size estimates, this time toward higher values. To get around this, we do not recommend simply lowering the variant calling threshold because NGS sequencing errors can also give rise to false positives, thereby inappropriately inflating bottleneck size estimates. Instead, we recommend accommodating below-threshold variants in the following way. For a donor-identified variant, *i*, that is called absent in the recipient (whether truly absent or just called absent), the likelihood of the transmission bottleneck size is given by equation 2, where *T* is the variant calling threshold (e.g., of 3%) and *p_beta_cdf*(ν_*R*,*i*_ < *T*|*k*, *N_b_* − *k*) is given by the beta cumulative distribution function evaluated at the variant calling threshold. We can again incorporate the effects of sampling noise by considering the number of reads at the variant site with equation 4, where, in this case, *p_betabin_cdf*(*R_var_*_,*i*_ < *TR_tot_*_,*i*_|*R_tot,i_*, *k*, *N_b_−k*) is given by the beta-binomial cumulative distribution function evaluated at the number of reads that would qualify as falling at the variant calling threshold.

Once the transmission bottleneck sizes have been estimated by using the beta-binomial sampling method, the probability of the true presence/absence of a variant in the recipient host can be determined for any given donor variant frequency. Similarly, the probability that a variant is called present/absent can be determined for any given donor frequency, ν_*D*,*i*_, given a sufficiently high read count in the recipient host. Given a high read count, the probability that a variant is called present in the recipient is given by $\overset{N_{b}}{\underset{k\;=\;0}{\sum }}\left[1\;-\;p\_beta\_cdf\;(v_{R,i}\;<\;T|k,\;N_{b}\;-\;k)]\;p\_bin\;(k\text{|}N_{b},\;v_{D,i}\right)$.

### The binomial sampling method.

In contrast to the beta-binomial sampling method, the binomial sampling method implicitly assumes that the infecting virus population is subject to deterministic dynamics between the time of infection and the time at which the recipient virus is sampled and, thus, that the sampled pathogen population in the recipient perfectly reflects the founding pathogen population under the common assumption of selective neutrality. The founding pathogen population is, as in the beta-binomial sampling method, assumed to be randomly sampled from the pathogen population of the donor host. The site-specific likelihood of the transmission bottleneck size, *N_b_*, is therefore given by equation 6, where p_bin (Rvar,i|Rtot,i, fk) = ()Rvar,iRtot,i (kNb)Rvar,i (1 − kNb)Rtot,i − Rvar,i. The overall likelihood of the transmission bottleneck size, *N_b_*, is calculated by multiplying across all site-specific likelihoods.

The above-described expression incorporates sampling noise, which is important when only a small number of reads are available. With an increasing number of reads, the sampling noise necessarily goes down, making *p_bin*(*R_var_*_,*i*_|*R_tot_*_,*i*_, *k*/*N_b_*) ≈ 0 in cases where *R_var_*_,*i*_/*R_tot_*_,*i*_ ≠ *k*/*N_b_*. This will result in dramatic differences in likelihood values between small values of *N_b_* and, more generally, multimodal likelihood curves that are very sensitive to specific variant frequencies in the recipient host.

One basic issue with this approach is therefore the assumption of where differences in variant frequencies across donor-recipient pairs stem from. Under this model, any observed differences are due to the presence of a transmission bottleneck because it assumes that the sampled pathogen population in the recipient perfectly reflects the founding pathogen population. This assumption is met under a scenario of deterministic, and neutral, viral population dynamics between the time of the transmission event and the time of pathogen sampling from the recipient host. For example, if we assume deterministic exponential growth from the time of the transmission event to the time of sampling, the dynamics of the viral population that carries the variant allele is given by *N*_ν_(*t*) = *N*_ν_(0)e^*rt*^, and similarly, the dynamics of the viral population that carries the reference allele is given by *N_r_*(*t*) = *N_r_*(0)*e*^*rt*^. At the time of the transmission event (*t* = 0), the fraction of the viral population that carries the variant allele is given by *k*/*N_b_*. At time *t*, the fraction of the viral population that carries the variant allele is given by *N*_ν_(*t*)/[*N*_ν_(*t*) + *N_r_*(*t*)], which simplifies to *k*/*N_b_*.

The bottleneck size estimates inferred with the binomial sampling method are again subject to the effects of false-negative variant calls. We can modify the binomial sampling method to incorporate the variant call threshold in a way similar to how the threshold frequency was incorporated into the beta-binomial sampling method. For a donor-identified variant, *i*, that is called absent in the recipient (whether truly absent or just called absent), the likelihood of the transmission bottleneck size is explained by equation 7. The probability that the number of variant reads falls below the level required for the variant to be called present is given by the binomial cumulative distribution function $p\_bin\_cdf\;(R_{\mathrm{var},i}\;<\;\left\lfloor {TR}_{tot,i}\right\rfloor |R_{tot,i},\frac{k}{N_{b}})\;=\;\sum_{j\;=\;0}^{\left\lfloor TR_{tot,i}\right\rfloor }\left(\underset{j}{R_{tot,i}}\right)\;(\frac{k}{N_{b}}{)}^{j}\;(1\;-\;\frac{k}{N_{b}}{)}^{R_{tot,i^{-\;j}}}$, where ⌊*TR*_*tot*,*i*_⌋ is the largest integer smaller than *TR*_*tot*,*i*_.

Once transmission bottleneck sizes have been estimated by using the binomial sampling method, the probability of the true presence/absence of a variant in the recipient host can again be determined for any given donor variant frequency. Similarly, the probability that a variant is called present/absent can be determined for any given donor frequency, ν_*D*,*i*_, provided information on the total read count in the recipient. Specifically, in the case of a high number of reads, the probability that a variant is called present (whether it is absent or present in the recipient host) is given by $\overset{N_{b}}{\underset{k\;=\;0}{\sum }}B\;(k,\;N_{b},\;T)\;p\_bin(k|N_{b},\;v_{D,i})$, where *B* (*k*, *N_b_*, *T*) is a Boolean function that evaluates to 1 if *k*/*N_b_* > *T* and 0 otherwise.

### Simulated deep-sequencing data.

To illustrate the use of the methods used to estimate *N_b_*, we generated a mock deep-sequencing data set via simulation. For this data set, we assumed a single donor-recipient pair, with 500 independent donor-identified variants. Independently for both the donor and the recipient, we drew the total number of reads at each of the 500 sites from a normal distribution with a mean of 500 reads and a standard deviation of 100 reads. Draws from the normal distribution were rounded to the nearest integer, and those that fell at 0 or below were discarded. For the donor, we then first determined “true” variant frequencies at each of these sites by drawing from an exponential distribution with a mean frequency of 0.08. Variants with observed frequencies below the variant calling threshold of 0.03 or above 0.50 were discarded. To determine the number of variant reads at a given site in the donor, we drew from a binomial distribution with the number of trials being the total read count at that site in the donor and the probability of success being given by that site's true variant frequency in the donor. We then incorporated sequencing error by again using draws from binomial distributions. Specifically, we determined the number of true reference reads in the donor that were misclassified as variant reads and the number of true variant reads in the donor that were correctly classified as variant reads, based on an assumed sequencing error rate of 1%. The total number of observed variant reads at a given site in a donor was then calculated as the sum of the misclassified reference reads and the correctly classified variant reads. Observed variant frequencies in the donor were then calculated by dividing the number of observed variant reads by the total number of observed reads at each site. In this manner, we simulated 500 variants, with observed frequencies in the range of 3 to 50%. The lower bound value of 3% was our assumed variant calling threshold; the upper bound value of 50% coincided with a variant allele always being the minority allele.

For the recipient, we simulated the total number of variant reads at each site by first simply determining, at each site, the number of virions in the founding population that carried the variant allele, under the assumption of a transmission bottleneck size, *N_b_*, of 50. This was done, at each site, by drawing from a binomial distribution with the number of trials being *N_b_* and the probability of success being the true variant frequency at that site in the donor. For the simulated data set, we first determined the true fraction of the viral population carrying the variant allele at the time of sampling by drawing from a beta distribution with the shape parameter being the number of variant alleles in the founder population and the scale parameter being the difference between the founding population size of *N_b_* and the number of variant alleles in the founder population. The true number of variant reads was then determined by drawing from a binomial distribution with the number of trials being the total number of reads at that site and the probability of success being the fraction of the population at the time of sampling that carried the variant allele. We then obtained the total number of variant reads at a given site in a recipient by introducing sequencing error to the true number of variant reads and the true number of reference reads.

### Application to influenza A virus deep-sequencing data.

We applied the three methods for bottleneck size inference described in the introduction to influenza A virus deep-sequencing data examined previously (28). In that study, Poon and colleagues identified donor-recipient transmission pairs based on household information and the genetic similarities between the viral populations in infected hosts. We base our analyses on these previously identified transmission pairs. In some cases, there were several members of a household who became infected. In this subset of cases, rather than considering all feasible pairwise combinations of who infected whom, we assumed that the index case transmitted the infection to the remaining household members. With this assumption, the 9 identified transmission pairs for influenza A virus subtype H1N1p were 681_V1(0) → 681_V3(2), 684_V1(0) → 684_V2(3), 712_V1(0) → 712_V1(4), 742_V1(0) → 742_V3(3), 751_V1(0) → 751_V3(1), 751_V1(0) → 751_V2(3), 751_V1(0) → 751_V2(4), 779_V1(0) → 779_V2(1), and 779_V1(0) → 779_V1(2), where *X*_V*Y*(*Z*) refers to household *X*, visit *Y*, and subject *Z* and the arrow demarcates transmission from the donor to the recipient. The 7 identified transmission pairs for influenza A virus subtype H3N2 were 689_V1(0) → 689_V2(2), 720_V1(0) → 720_V2(1), 734_V1(0) → 734_V3(2), 739_V1(0) → 739_V2(2), 739_V1(0) → 739_V2(3), 747_V1(0) → 747_V2(2), and 763_V1(0) → 763_V2(3). The deep-sequencing data are publically available (28) (see https://www.synapse.org/#!Synapse:syn8033988). We called variants and determined variant frequencies from these data using VarScan (45, 46), using a variant calling threshold of 3%, a mean quality score of 20, and a *P* value of 0.05. We provide variants and their frequencies used in this study in Data Set S1 in the supplemental material.

### Calculation of overall transmission bottleneck sizes across transmission pairs.

To calculate transmission bottleneck sizes over multiple transmission pairs, we did not take simply the sum of log likelihoods across transmission pairs. Taking simply the sum would inappropriately give greater weight to transmission pairs with a larger number of donor-identified variants. To weight each of the transmission pairs equally, we scaled the log likelihood of each transmission pair based on the number of variants identified in that transmission pair, such that the overall log likelihood was given by $\sum_{p\;=\;1}^{N}\frac{n_{\max}}{n_{p}}\;\log L_{p}\;(N_{b})$, where *N* is the number of transmission pairs, *n_p_* is the number of donor-identified variants in transmission pair *p*, *n*_max_ equals max(*n_p_*), and logL*_p_*(*N_b_*) are the log likelihoods across *N_b_* values in transmission pair *p*.

## Supplementary Material

Supplemental material
