# Correction for Sobel Leonard et al., “Transmission Bottleneck Size Estimation from Pathogen Deep-Sequencing Data, with an Application to Human Influenza A Virus”

**DOI:** 10.1128/JVI.00936-19

**Published:** 2019-08-13

**Authors:** Ashley Sobel Leonard, Daniel B. Weissman, Benjamin Greenbaum, Elodie Ghedin, Katia Koelle

**Affiliations:** a Department of Biology, Duke University, Durham, North Carolina, USA; b Department of Physics, Emory University, Atlanta, Georgia, USA; c Tisch Cancer Institute, Departments of Medicine, Oncological Sciences, and Pathology, Icahn School of Medicine at Mount Sinai, New York, New York, USA; d Center for Genomics and Systems Biology, Department of Biology, and College of Global Public Health, New York University, New York, New York, USA

## AUTHOR CORRECTION

Volume 91, no. 14, e00171-17, 2017, https://doi.org/10.1128/JVI.00171-17. As an application of the transmission bottleneck size estimation method developed in this paper, we used a previously published influenza A data set first presented by L. L. M. Poon, T. Song, R. Rosenfeld, X. Lin, et al. [Nat Genet 48(2):195-200, 2016, https://doi.org/10.1038/ng.3479]. Recently, K. S. Xue and J. D. Bloom (Nat Genet, 25 February 2019, https://doi.org/10.1038/s41588-019-0349-3) have shown that the Poon et al. data set is “technically contaminated” with read pairs split between unrelated samples, which had the effect of inflating the similarities in allele frequencies between samples. As a result, when we applied our betabinomial approach to the Poon et al. data set, it yielded transmission bottleneck size estimates that are incongruous with, and larger than, other transmission bottleneck size estimates for seasonal influenza A virus. The validity of the betabinomial estimation method presented in our paper is itself unaffected. While we therefore continue to encourage the use of our developed estimation method on other data sets, we would like to caution the reader against citing our paper as providing evidence for a loose transmission bottleneck size for influenza A virus.

Computer code for the betabinomial transmission bottleneck size estimation method is available on GitHub at https://github.com/koellelab/betabinomial_bottleneck.

