# Supplementary material for: Transmission Bottleneck Size Estimation from Pathogen Deep-Sequencing Data, with an Application to Human Influenza A Virus
Source: J Virol. 2017 Jun 26;91(14):e00171-17. doi: 10.1128/JVI.00171-17 (PMC5487570; doi:10.1128/JVI.00171-17)
Supplement: Supplemental material [file supp_91_14_e00171-17__index.html]

Transmission Bottleneck Size Estimation from Pathogen Deep-Sequencing Data, with an Application to Human Influenza A Virus — Supplemental material 

# Transmission Bottleneck Size Estimation from Pathogen Deep-Sequencing Data, with an Application to Human Influenza A Virus

## Supplemental material

- Supplemental file 1 -

  Data Set S1 (Variants identified in the donor-recipient transmission pairs.)

  XLSX, 80K
